# Targeting patients for early COVID-19 therapy; Pre-infection metabolic dysfunction, polycystic ovary syndrome and risk of severe disease in patients under 65: A Massachusetts community-based observational study

**DOI:** 10.1371/journal.pone.0287430

**Published:** 2023-06-15

**Authors:** Susan R. Sama, Rebecca Gore, Ann Z. Bauer, Lawrence Garber, Richard Rosiello, Devi Sundaresan, Anne McDonald, David Kriebel

**Affiliations:** 1 Department of Public Health, University of Massachusetts Lowell, Lowell, Massachusetts, United States of America; 2 Reliant Medical Group, Inc., Worcester, Massachusetts, United States of America; 3 Great Meadows Public Health Collaborative, Wayland, Massachusetts, United States of America; The University of Lahore, PAKISTAN

## Abstract

**Introduction:**

The demographics of those developing severe coronavirus disease (COVID-19) outcomes are shifting to younger patients. In an observational study utilizing electronic health records from a Massachusetts group medical practice, we identified 5025 patients with confirmed COVID-19 from March 1 to December 18, 2020. Of these, 3870 were under 65 years of age. We investigated the hypothesis that pre-infection metabolic or immunologic dysregulation including polycystic ovary syndrome (PCOS) increased risk of serious COVID-19 outcomes in patients under 65 years of age.

**Materials and methods:**

We compared those with COVID-19 related hospitalization or mortality to all other COVID-19 patients, using a case control approach. Using logistic regression and propensity score modeling, we evaluated risk of developing severe COVID-19 outcomes (hospitalization or death) in those with *pre-infection* comorbidities, metabolic risk factors, or PCOS.

**Results:**

Overall, propensity score matched analyses demonstrated *pre-infection* elevated liver enzymes alanine aminotransferase (ALT) >40, aspartate aminotransferase (AST) >40 and blood glucose ≥215 mg/dL were associated with more severe COVID-19 outcomes, OR = 1.74 (95% CI 1.31, 2.31); OR = 1.98 (95% CI 1.52, 2.57), and OR = 1.55 (95% CI 1.08, 2.23) respectively. Elevated hemoglobin A1C or blood glucose levels were even stronger risk factors for severe COVID-19 outcomes among those aged < 65, OR = 2.31 (95% CI 1.14, 4.66) and OR = 2.42 (95% CI 1.29, 4.56), respectively. In logistic regression models, women aged < 65 with PCOS demonstrated more than a four-fold increased risk of severe COVID-19, OR 4.64 (95% CI 1.98, 10.88).

**Conclusion:**

Increased risk of severe COVID-19 outcomes in those < age 65 with *pre-infection* indicators of metabolic dysfunction heightens the importance of monitoring pre-infection indicators in younger patients for prevention and early treatment. The PCOS finding deserves further investigation. Meanwhile women who suffer from PCOS should be carefully evaluated and prioritized for earlier COVID-19 treatment and vaccination.

## Introduction

Severe acute respiratory syndrome from coronavirus 2 (SARS-CoV-2) is a significant global public health threat which has multiple pathophysiologic metabolic interconnections. While advanced age is a known risk factor, severe outcomes are increasingly occurring in younger patients and information about this population is lacking [1, 2]. Pre-existing comorbidities associated with impaired glucose metabolism, as well as hormone or hepatic abnormalities increase risk of hospitalization or death from SARS-CoV-2 infection [3–6]. Metabolic dysfunction including diabetes is a known risk factor for severe coronavirus disease (COVID-19) outcomes and risk associated with increased blood glucose levels and abnormal liver biochemistry has recently been under investigation, as well [7–11]. Several investigators have proposed that women of reproductive age with polycystic ovary syndrome (PCOS) are potentially at higher risk of severe COVID-19 outcomes, because many clinical findings in PCOS are risk factors for COVID-19 [12–17].

Diabetes and markers of *pre-infection* glycemic control (A1C and blood glucose levels) have been associated with poor outcomes of COVID-19 including mortality and increased risk of hospitalization [18–21]. The aminotransferases alanine transaminase (ALT) and aspartate aminotransferase (AST) are enzymes the liver uses to make glycogen, a stored form of glucose. More than one third of patients admitted to the hospital with SARS-CoV-2 infection have abnormal liver function and longer hospital stays [22]. Studies have also shown that abnormal laboratory indicators of liver injury including elevated ALT and AST levels, are associated with increased COVID-19 related morbidity and mortality [23–27].

PCOS is the most frequent endocrine disorder in women of reproductive age, diagnosed in up to 20% of women [28, 29]. The syndrome is associated with the following: hyperandrogenism, obesity, diabetes mellitus, dyslipidemia, hypertension, and non-alcoholic fatty liver disease, which can all significantly increase risk of adverse COVID-19 related outcomes [13, 30–39]. Subramanian and colleagues reported that risk of contracting SARS-CoV-2 was higher in women with PCOS compared to women without PCOS (HR: 1.28; 1.05–1.56) [39]. Because PCOS is associated with these metabolic outcomes, we hypothesized that PCOS puts women infected with SARS-CoV-2 at increased risk of severe COVID-19 [13, 17, 39–43]. While there have been several studies proposing this hypothesis there is a paucity of epidemiologic research [12–17, 39].

We have expanded a previously-published study [44] of a community-based cohort now including 5025 patients, of whom 3870 were under age 65, and diagnosed with COVID-19 in central Massachusetts, USA. We were interested in investigating the hypothesis that pre-infection metabolic or immunologic dysregulation may increase risk of severe COVID-19 outcomes (defined in this study as hospitalization or death), particularly in those under 65 years of age. We investigated whether patients with elevated glycated hemoglobin (A1C), blood glucose, ALT or AST, and women with PCOS were at increased risk of severe COVID-19, while controlling for potential confounding by co-morbidities.

## Materials and methods

### Study design

In this observational case–control study we used de-identified administrative claims and electronic health record (EHR) data from a large comprehensive health care system serving central Massachusetts USA, during the study period of March 1 to December 18, 2020. In this investigation we followed similar data extraction and analysis methods as in our earlier investigation of hypertension, medications and risk of severe COVID-19 [44].

### Characteristics of patients

We identified 5025 hospitalized and non-hospitalized patients, 18 to 106 years old with a positive reverse transcription polymerase chain reaction (RT-PCR) test (CPT codes: 87635.**, 87798.189) or an ICD-10 Code of U07.1 indicating a COVID-19 diagnosis between March 1 and December 18, 2020 [44]. This COVID-19 diagnosis was based either on PCR test or symptoms, following CDC guidelines [45].

For each identified patient with a COVID-19 diagnosis, we used medical history data from the EHR prior to the incident date of their first positive COVID-19 test—including encounter diagnosis codes, medication order data, lab tests and results, problem lists and demographics (gender, age, smoking status, body mass index (BMI)) [44]. Prior information for each health indicator closest to the first positive covid test was used. Hereafter we refer to this as the “*pre-infection*” period.

### Definitions of cases and controls, exposures and outcomes

All identified COVID-19 patients were divided into two groups based on severity of COVID-19 and compared using a case control approach. “Cases” in our study were all individuals with severe COVID-19 defined by COVID-19-related hospitalization or death, while the remaining COVID-19 positive patients constituted the “controls” and were designated as “Not Severe” [44]. Hospitalizations were considered COVID-19-related if they occurred within the 3-week period after having been diagnosed with COVID-19; or if there was a positive COVID-19 test while a person was hospitalized, within a week of the hospitalization. A hospitalization outside of this COVID-19 test window was not considered COVID-19 related, and therefore not a severe case. Deaths were considered COVID-19 related if they occurred within 90 days after the COVID-19 diagnosis. Data on severity of COVID-19 were obtained from the medical record at the moment of enrollment into the study.

Encounter and diagnostic codes prior to COVID-19 diagnosis were used to characterize patients as having hypertension (HT), diabetes, chronic respiratory disease, congestive heart failure, immunosuppressed conditions (HIV or history of solid organ transplant), chronic kidney disease, chronic liver disease, cancer, and PCOS. The most recent lab tests prior to COVID-19 diagnosis were abstracted, to ascertain exposure to high A1C, blood glucose, ALT, and AST. Eighty percent of these were collected within 12 months prior to COVID-19 diagnosis and all within 23 months. (See S1 Appendix: Description of Study Codes and Algorithms.) Standard clinical guidelines were used to define cutpoints determining high exposure levels. (See S1 Appendix: Description of Study Codes and Algorithms.) The potential confounding variables of gender, age, smoking status, body mass index (BMI) were also extracted from the EHR. To improve accuracy, we used the last informative value prior to COVID-19 diagnosis to capture smoking status and BMI. In a prior study of this patient population, we conducted a subset of manual chart reviews to develop our extraction algorithm, to conduct data cleaning and to validate proper categorization of severity [44].

### Data sources and measurement

The data source for all variables was the EHR. (See S1 Appendix: Description of Study Codes and Algorithms.) All methods were performed in accordance with the relevant guidelines and regulations. This study included only adults (age 18 and older) and was reviewed and approved via expedited review by the Institutional Review Board of the University of Massachusetts Lowell: IRB number: 20–055 with a waiver of informed consent.

### Statistical analysis

Differences in demographics and pre-existing conditions between severe and not-severe COVID-19 patients were compared using the Chi-square for categorical data or Wilcoxon tests for continuous data. Logistic regression models were then fit to evaluate COVID-19 severity and pre-infection levels of A1C, blood glucose ALT, AST, and PCOS diagnosis (women only) adjusted for the potential confounders of age and gender. BMI was imputed for those patients for whom it was missing (8.9%), using the overall study population median BMI of 30.1 (SD 7.4).

Because of the very strong effect of older age on COVID-19 severity and emerging data showing that younger patients are increasingly suffering higher rates of severe disease [1], we used logistic regression to estimate the associations between COVID-19 severity and the same set of pre-existing comorbidities in those younger than 65 years.

After initial multiple logistic regression models identified high A1C, high blood glucose, ALT, AST and diagnosis of PCOS (women only) as risk factors, we turned to propensity score matching to provide tighter control over multiple confounders and potential selection bias [46]. The first step in using propensity scores was to construct a logistic regression model to predict the probability of an individual having each of the risk factors of interest, within the study population [44].

The candidates for predictors were 12 factors found in initial univariate logistic regression models to be associated with the metabolic and hepatic indicators of interest, as well as their two-way interactions: age, gender, BMI, race, ethnicity, systolic BP, diabetes, chronic respiratory disease, chronic kidney failure, arterial disease, hypertension, and congestive heart failure. When models including the full set of potential predictors and all two-way interactions failed to converge, terms were dropped, until convergence was achieved. The largest subset of all possible interactions was included in each final model. (See S2 Appendix: Model Composition and Sensitivity Analyses.) We defined the propensity score by using the predicted values from the logistic regression, which represent the probability that someone is exposed to the risk factor (high A1C, high blood glucose, ALT and AST >40, and PCOS) of interest [44]. A similar procedure was followed for the propensity score models in the analyses restricted to only those under age 65. Because younger patients have fewer comorbidities and less abnormal blood tests, these models typically included fewer predictors compared to models run on the full cohort.

In both the overall and < age 65 analyses, each exposed subject was matched (using Greedy Matching) to up to three unexposed subjects without replacement having the closest available propensity scores [46]. Matched sets never differed by more than 0.25 of the standard deviation of the propensity scores as recommended by Rosenbaum and Rubin [47]. In some cases, three unexposed subjects did not meet this criterion in which case only two or one were selected. Conditional logistic regression was then used to calculate the hazard ratio (equivalent to the conditional OR in these case control data) for risk of severe COVID-19 from the dichotomous exposure variable within propensity score matched sets. We repeated this procedure with all the different risk factors. For validation purposes we also calculated the absolute standard mean differences of the demographic variables in the propensity score models. All statistical analyses were conducted using SAS 9.4 (SAS Institute Inc., Cary, NC, USA) [44].

## Results

**Fig 1:**
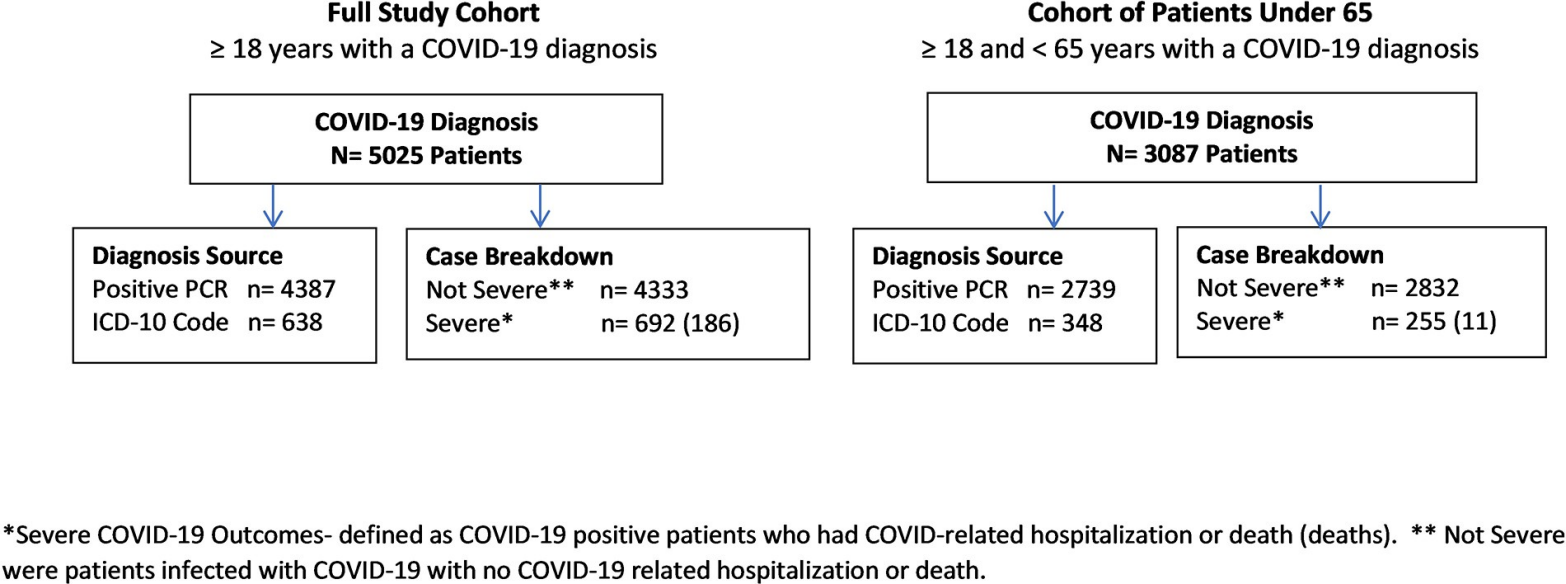
Flow diagrams of patients overall and under age 65 by severe COVID-19 outcomes.

As of December 2020, we identified 5025 COVID-19 patients in the central Massachusetts group medical practice (Table 1). In our cohort, 4387 patients had a positive RT-PCR laboratory test (87%) and 638 were clinically confirmed COVID-19 cases (13%) (Fig 1). There were 3870 patients < age 65, of whom 91% had a positive PCR test. Overall, 692 (14%) were categorized as severe; 506 (10%) were hospitalized and did not die, while 186 (4%) died. Of the 3870 patients under age 65, 255 (7%) were categorized as severe; 244 were hospitalized and did not die, while 11 (0.3%) died. Women made up almost 60% of the cohort, which is consistent with Massachusetts data showing that more women were diagnosed with COVID-19 than men. (41) More severe cases on average were older than the less severe (70 vs 47 years, respectively). Mean systolic and diastolic blood pressures were within normal range for both severe and non-severe patients.

**Table 1:** Demographics, baseline characteristics of severe COVID-19.

All COVID-19 Patients	Total COVID-19 Patients	Not Severe* COVID-19	Severe** COVID-19	Test of DifferenceƗ
	(N = 5025)	4333 (86.2%)	692 (13.8%)	
Males	2090 (42%)	1773 (41%)	317 (46%)	0.0154
Age (mean, s.d.)	50.4 (20.4)	47.3 (18.8)	69.8 (19.3)	< .0001
BMI (N = 4459)	30.1 (7.4)	30.0 (7.3)	30.7 (7.9)	0.116
Race				
White	2952 (58.8%)	2512 (58.0%)	440 (63.6%)	
Black	326 (6.5%)	289 (6.7%)	37 (5.4%)	
Unknown	1447 (28.8%)	1265 (29.2%)	182 (26.3%)	
Other	300 (6.0%)	267 (6.2%)	33 (4.8%)	0.037
Ethnicity				
Hispanic	492 (9.8%)	452 (10.4%)	40 (5.8%)	
Unknown	1964 (39.1%)	1695 (39.1%)	269 (38.9%)	0.0003
Ever Smoker Y/N (n = 4700)	1673 (35.6%)	1373 (33.7%)	300 (47.7%)	< .0001
Current Smoker	326 (6.5%)	294 (6.8%)	32 (4.6%)	
Former Smoker	1347 (26.8%)	1079 (24.9%)	268 (38.7%)	
Never Smoker	3027 (60.2%)	2698 (62.3%)	329 (47.5%)	
Unknown Smoking Status	325 (6.5%)	262 (6.0%)	63 (9.1%)	< .0001
Systolic pressure (mean, s.d.)	(123.6, 15.5)	(123.2, 15.1)	(127.2, 17.3)	< .0001
Diastolic pressure (mean, s.d.)	(75.6, 9.1)	(75.9, 9.0)	(73.8, 9.6)	< .0001
Resident of Long-Term Care Y/N	418 (8.3%)	251 (5.8%)	167 (24.1%)	< .0001

Abbreviations: s.d, standard deviation **Ɨ**p-value testing H0: no difference between severe and not severe COVID-19 patients. Chi-square test (categorical data) and Wilcoxon (continuous data).

* Not severe were patients infected with SARS-CoV-2 with no COVID-19 related hospitalization or death.

**Severe: defined as COVID-19 positive patients who had a COVID-related hospitalization or death.

Initial logistic regression models controlling for age and gender showed HT, diabetes, chronic respiratory disease, arterial disease, congestive heart failure, chronic kidney disease, cancer, and PCOS were associated with more severe COVID-19 (Table 2). In COVID-19 patients under 65 years of age, HT, diabetes, high BMI, arterial disease, congestive heart failure, chronic renal disease, and cancer were even more strongly associated with increased risk of severe COVID-19 than in our overall study population (Table 2). In females (< age 65), PCOS demonstrated over a four-and-a-half-fold increase in risk of severe COVID-19 (OR = 4.64, 95% CI 1.98, 10.88).

**Table 2:** Logistic regression analyses of comorbidities and severe COVID-19.

	All Participants			Under 65 Years	
	N = 5025			N = 3870	
	Overall	OR** (95% CI)	Adj OR*** (95% CI)	Overall	Adj OR*** (95% CI)
Comorbidities	Prevalence	Severe COVID-19*	Severe COVID-19*	Prevalence	Severe COVID-19*
Hypertension	1436 (28.6%)	4.27 (3.61, 5.04), p<0.0001	1.42 (1.17, 1.73), p<0.0004	681 (17.6%)	2.05 (1.53, 2.75), p<0.0001
Diabetes Mellitus	673 (13.4%)	4.33 (3.60, 5.22), p<0.0001	2.04 (1.66, 2.50), p<0.0001	309 (8.0%)	2.86 (2.06, 3.98), p<0.0001
BMI_High Ɨ Ɨ (>30)	593 (13.3%)	1.14 (0.95, 1.35), p = 0.153	1.36 (1.12, 1.66), p = 0.0017	1570 (44.4%)	1.95 (1.48, 2.58), p<0.001
Chronic Resp. Disease	1058 (21.1%)	1.79 (1.50, 2.14), p<0.0001	1.51 (1.24, 1.84), p<0.0001	722 18.7%)	1.50 (1.12, 2.02), p = 0.0071
Arterial Disease	290 (5.8%)	5.66 (4.42, 7.26), p<0.0001	1.51 (1.14, 1.99), p = 0.0039	59 (1.5%)	2.05 (1.06, 3.98), p = 0.0332
Congestive Heart Failure	211 (4.2%)	9.37 (7.05, 12.47), p<0.0001	2.39 (1.75, 3.28), p<0.0001	22 (<1%)	7.79 (3.26, 18.65), p<0.0001
Immunosuppressed	61 (1.2%)	2.06 (1.15, 3.72), p = 0.0157	1.11 (0.57, 2.15), p = 0.7585	33 (<1%)	2.35 (0.88, 6.23), p = 0.0873
Chronic Renal Disease	446 (8.9%)	6.83 (5.54, 8.42), p<0.0001	1.75 (1.37, 2.23), p<0.0001	66 (1.7%)	3.65 (2.06, 6.45), p<0.0001
Cancer	304 (6.1%)	3.72 (2.89, 4.78), p<0.0001	1.53 (1.16, 2.02), p = 0.0025	121 (3.1%)	2.35 (1.44, 3.82), p = 0.0006
Chronic Liver Disease	48 (1.0%)	2.11 (1.09, 4.07), p = 0.0266	1.85 (0.93, 3.70), p = 0.0817	32 (<1%)	1.39 (0.48, 4.06), p = 0.5429
Polycystic Ovary Syndrome^Ɨ^	52 (1.8%)	1.25 (0.58, 2.67), p = 0.5703	5.18 (2.29, 11.73), p<0.0001	51 (2.3%)	4.64 (1.98, 10.88), p = 0.004

*Severe COVID-19—defined as COVID-19 positive patients who had a COVID-related hospitalization or death.

**OR = odds ratio

***Adj OR = Adjusted odds ratio controlled for age and gender, Ɨ Polycystic Ovary Syndrome in females, under 65 years of age; Ɨ Ɨ BMI only available in 4459 patients overall and 3536 patients under 65.

In the propensity score models, the calculated absolute standard mean differences of the demographic variables including age, BMI, systolic BP, and gender were small, none larger than 0.199. This suggests propensity score matching resulted in groups that were similar on important covariates.

Utilizing propensity score models, pre-infection high blood glucose (OR = 1.55, 95% CI 1.08, 2.23) was associated with severe COVID-19 outcomes in the all ages cohort. Patients with pre-infection ALT >40 (OR = 1.74, 95%CI 1.31, 2.31) and AST >40 (OR = 1.98, 95%CI 1.52, 2.57) were also at increased risk of severe COVID-19. Pre-infection A1C did not demonstrate increased risk of severe disease, (OR = 1.34, 95%CI 0.82. 2.41) (Table 3).

**Table 3:** All ages: Pre-infection clinical characteristics and risk of severe COVID-19.

Exposures	Severe COVID-19*		
	Odds Ratio	95% Confidence Interval	p-value
A1C high (> = 9) All ages	OR = 1.34	(0.82, 2.17)	0.24
Blood Glucose High (> = 215 mg/dL) All ages	OR = 1.55	(1.08, 2.23)	0.02
ALT > 40 All Ages	OR = 1.74	(1.31, 2.31)	<0.0001
AST >40 All Ages	OR = 1.98	(1.52, 2.57)	<0.0001

* Severe COVID-19- defined as COVID-19 positive patients who had a COVID-related hospitalization or death. Each exposure was matched (using Greedy Matching) to up to three unexposed subjects having the closest available propensity scores, without replacement.

**Fig 2:**
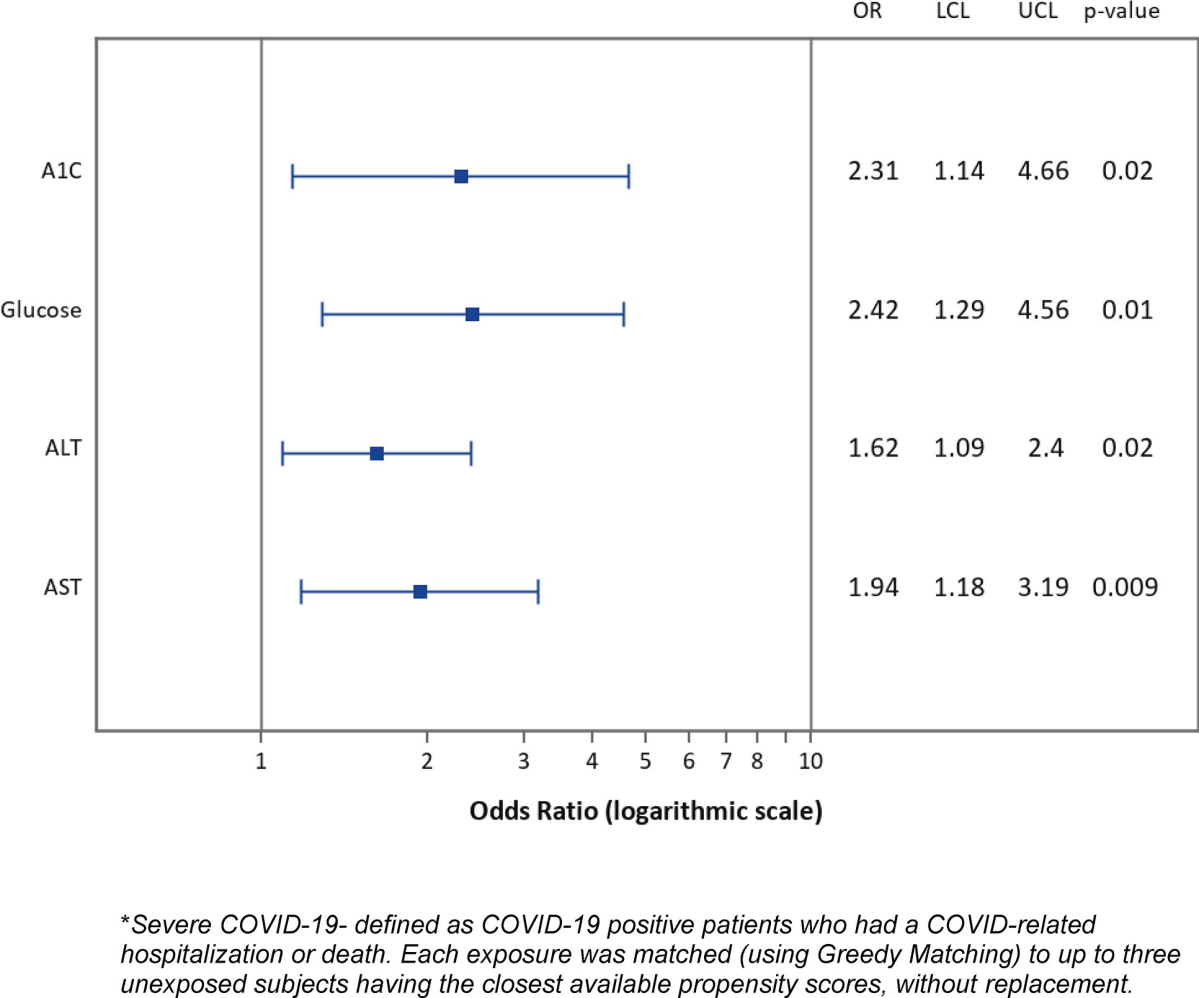
Under 65: Pre-infection clinical characteristics and risk of severe COVID-19.

Propensity score models also demonstrated that patients < age 65 with high pre-infection A1C and blood glucose demonstrated even greater risk of severe COVID-19 outcomes, (OR = 2.31, 95% CI 1.14, 4.66) and (OR = 2.42, 95% CI 1.29, 4.56), respectively compared to patients overall (Fig 2). As our data included only 52 women with PCOS, propensity score models for this exposure were imprecise and yielded wide confidence intervals. Nevertheless, the point estimate for the association PCOS–severe COVID-19 (OR = 4.2, 95%CI 1.33, 13.23) was in good agreement with the logistic regression result.

Overall, 637 patients (13%) and in those < age 65, 348 patients (9%) were clinically diagnosed with COVID-19 but had no PCR test. We conducted a sensitivity analysis excluding clinically diagnosed patients and the results were substantively the same. (See S2 Appendix: Model Composition and Sensitivity Analyses.) For example, for one of our primary hypotheses, the effect of high blood glucose level overall, the odds ratio changed from 1.55 (1.08, 2.23) to 1.73 (1.16, 2.57).

## Discussion

In a previous publication, we reported on the relationship between anti-hypertensive medication use and risk of severe COVID-19 in a cohort of patients from a large group medical practice in central Massachusetts [44]. For the current study, we increased the size of the cohort, by assessing a longer time period, enabling us to investigate the relationship between indicators of metabolic dysfunction and COVID-19 outcomes overall and in those under age 65. We focused on pre-infection A1C, blood glucose, liver aminotransferases ALT and AST, and PCOS among all individuals diagnosed with COVID-19 in this community-based population.

Early in the pandemic, diabetes was found to be closely correlated with increased risk of COVID-19 and with poor clinical outcomes [48, 49]. Angiotensin converting enzyme 2 (ACE2) is a receptor that facilitates entry of SARS-CoV-2 into cells and ACE2 has been shown to be overexpressed in those with diabetes mellitus compared to those without diabetes [50, 51]. We suspect that in our study, indicators of metabolic dysfunction also identify pre-diabetic patients, who likely share this ACE2 phenotypic trait [43]. Our findings are consistent other studies indicating increased risk of more serious or severe COVID-19 outcomes in patients with high A1C [52, 53] and blood glucose [7, 8, 10, 11, 54, 55] and underline the importance of monitoring these pre-infection indicators in younger patients.

Our finding that patients with elevated pre-infection ALT and AST levels are also at increased risk of severe COVID-19 may be important for early identification of high-risk individuals, as ALT and AST measurements are components of widely administered comprehensive metabolic panel screening tools. While others have shown that liver injury in COVID-19 patients upon or during hospital admission is associated with increased morbidity and mortality [24, 26, 56–58], we believe we are the first to show that patients with elevated *pre-infection* ALT and AST levels are also at increased risk of severe COVID-19. Interestingly, we found that a clinical diagnosis of chronic liver disease was not associated with severe COVID-19; which is consistent with the pooled analysis done by Lippi et al. [59]. One possible explanation for this paradox is that hepatocellular damage may result in the elevation of serum AST and ALT levels in early onset liver disease [9]. It has been proposed that elevated transaminase levels identified in hospitalized COVID-19 patients could develop from hepatocellular injury from ischemia, hypoxia, severe inflammation, or an immune response to a cytokine storm [60, 61]. In contrast pre-infection elevated AST or ALT may indicate early onset hepatocellular damage that predisposes to development of these conditions. Our findings suggest the elevated levels of ALT and AST could be utilized to prioritize patients for vaccinations, vaccine boosters and earlier treatments, as new modalities evolve.

We believe this study is the first to have observational data to support the suggestion that women suffering from PCOS could be at higher risk for severe COVID-19 [15, 16, 39]. In women of reproductive age, PCOS is the most frequent endocrine disorder with a prevalence which may reach or even exceed 10–15% [62]. Its prevalence is markedly higher in African American and South Asian women compared to white women. The prevalence of PCOS in this cohort was considerably lower (1.8%), perhaps because our catchment area is largely white, and possibly also due to underreporting in the EHR data [63]. PCOS occurs in younger women, an age group that is at relatively low risk of severe COVID-19. However, PCOS is associated with obesity, insulin resistance, hyperandrogenism, type 2 diabetes mellitus and being part of certain ethnic minorities, all of which have been shown to increase risk of developing severe COVID-19 [12–17, 64]. Despite these complex interactions among risk factors, the propensity score model results remained robust after controlling for BMI and diabetes, which supports the hypothesis that PCOS may present its own inherent risk. Optimal management of women suffering from PCOS requires a focused approach by the healthcare community. We recommend primary care and medical specialists caring for women with PCOS document and implement specialized care plans to address the increased risk these women face from COVID-19.

Strengths of our study include: EHR data from a well-studied community-based population, and chart reviews to verify case status and other study variables. Our study is limited by being observational, however we conducted a propensity score matched analysis, a highly effective method to reduce biases when estimating exposure effects [65]. We used *pre-infection* levels of metabolic dysfunction indicators prior to COVID-19 diagnosis and these measures can change over time. We relied on proper coding of comorbidities, as these codes are extracted from the EHR. In addition, we categorized COVID-19 severity at one point in time. Some patients categorized as mild could have progressed to severe after our dataset was obtained. And even though we attempted to verify cause of death with chart reviews, some deaths may have been misclassified as COVID-related. This would not be the case for our PCOS findings as none of these women died. We doubt that this would have introduced important systematic errors, however, misclassification bias and uncontrolled confounding cannot be completely ruled out. While propensity score modeling can improve both internal and external validity, generalization of our results should be made with caution, as our cohort may not be representative of the broader population [66].

## Conclusions

Our findings suggest that pre-infection indicators of metabolic disorders, elevated A1C and blood glucose levels, liver enzymes ALT and AST >40 and a comorbidity of PCOS in women may be useful predictors of susceptibility to severe COVID-19, even in younger patients. These early indicators could prove useful for identifying the vulnerable so that proactive precautions can be taken at an individual level including vaccination, vaccine boosters and masking. Population level interventions such as high air exchange ventilation, air filtration and germicidal UV light would also help protect this population [67]. Those infected with SARS-CoV-2 who present with these pre-existing indicators and comorbidities should be assessed for the need for early treatments such as monoclonal antibodies and antivirals, regardless of age. These treatments have been shown to reduce the likelihood of severe COVID-19 outcomes [68, 69]. These findings merit further investigation in other populations.

## Supporting information

S1 Appendix Description of study codes and algorithms. (DOCX)

S2 Appendix Model composition and sensitivity analyses. (DOCX)

S3 Appendix RECORD checklist final. (DOCX)

## List of abbreviations

A1C: Hemoglobin A1C
ACE2: Angiotensin converting enzyme 2
ALT: Alanine aminotransferase
AST: Aspartate aminotransferase
BMI: Body Mass Index
BP: Blood pressure
COVID-19: Coronavirus Disease 2019
EHR: Electronic Health Record
HT: Hypertensio
OR: Odds ratio
PCOS: Polycystic Ovary Syndrome
PCR: Polymerase chain reaction
RT-PCR: Reverse transcription polymerase chain reaction
SARS: CoV-2-Severe acute respiratory syndrome coronavirus 2
