# Supplementary material for: Targeting patients for early COVID-19 therapy; Pre-infection metabolic dysfunction, polycystic ovary syndrome and risk of severe disease in patients under 65: A Massachusetts community-based observational study
Source: PLoS One. 2023 Jun 15;18(6):e0287430. doi: 10.1371/journal.pone.0287430 (PMC10270632; doi:10.1371/journal.pone.0287430)
Supplement: S1 Appendix — (DOCX) [file pone.0287430.s001.docx]

**S1 Appendix: Description of Study Codes and Algorithms**

**Identification of COVID Patients for Study Sample:** CPT Codes for SARS-COV-2 reverse transcription polymerase chain reaction, RT-PCR ( 87635.** and 87798.189) with positive result and ICD-10 Code (U07.1) were used by the RMG data analyst to extract patients with a COVID-19 diagnosis. (Tables 1 and 2)

| **Table S1A. Procedure Codes for Identifying Patients with COVID-19** | |
| --- | --- |
| **Procedure Code** | **Procedure Name** |
| 87635.** | SARS CORONAVIRUS W/COV2 RNA, QL RT-PCR |
| 87798.189 | SARS-COV-2 RNA, QUALITATIVE REAL-TIME RT-PCR |

| **Table S1B. ICD-10 Code for Identifying Patients with COVID-19** | |
| --- | --- |
| **ICD-10 Code** | **Description** |
| U07.1 | COVID-19 |

**Outcome:** All identified COVID-19 patients were divided into two groups based on severe COVID-19 outcomes[1], comparing those with hospitalization or mortality to all other COVID-19 patients, using a case control approach. “Cases” in our study were all individuals with severe COVID outcomes defined by COVID-related hospitalization or death, while the remaining COVID-19 positive patients constituted the “controls” and are designated as “Not Severe”. Hospitalizations were considered COVID-related if they occurred within the 3-week period after having been diagnosed with COVID; or if there was a positive COVID-test while a person was hospitalized, within a week of the hospitalization. A hospitalization outside of this COVID-test window was not considered COVID-related, and therefore not a severe case. Deaths were considered COVID-related if they occurred within 90 days after the COVID-diagnosis. Data on severity were obtained from the medical record at the moment of enrollment into the study.

**Confounders and Effect Modification:** We evaluated the confounding effects of age (continuous) and gender (categorical) in our logistic regression models. Effect Modification was not evaluated in our logistic regression modeling.

For the propensity scored models the candidates for predictors were 12 factors found in initial univariate logistic regression models to be associated with the metabolic and hepatic indicators of interest, as well as all two-way effect modifiers: age, gender, BMI, race, ethnicity, systolic BP, diabetes, chronic respiratory disease, chronic kidney failure, arterial disease, hypertension, and congestive heart failure. When models including the full set of potential predictors failed to converge, terms were systematically dropped, until convergence was achieved.

In both the overall and age restricted analyses, each exposed subject was matched (using Greedy Matching) to up to three unexposed subjects without replacement having the closest available propensity scores. [2] Matched sets never differed by more than 0.25 of the standard deviation of the propensity scores as recommended by Rosenbaum and Rubin. [3] In some cases, three unexposed subjects did not meet this criterion in which case only two or one were selected. Conditional logistic regression was then used to calculate the hazard ratio (equivalent to the conditional OR in these case control data) for risk of severe COVID-19 from the dichotomous exposure variable within propensity score matched sets. We repeated this procedure with all the different risk factors.

**Diagnosis and Procedure Codes:** ICD-10 codes associated with encounter data (prior to first COVID-19 diagnosis) were used to categorize presence or absence of comorbidities (Table 3). Procedure codes (CPT codes) and component names were used extract lab tests and results and for the following variables:

- Hemoglobin A1C: procedure codes starting with 83036 or 83037 and component name having A1C. Cut point for high >=9.
- Blood Glucose: procedure codes starting with 83036 or 83037 and component name having blood glucose. Cut point for high >= 215 mg/dL.
- Alanine transaminase (ALT): Component name starts with ALT. See Table 5 for procedure codes. Cut point > 40.
- Aspartate aminotransferase (AST): Component name starts with AST. See Table 6 for procedure codes. Cut point > 40.

Demographic variables including age, gender, race, ethnicity, BMI, smoking status were also extracted from the Electronic Health Record. In order to improve accuracy, we extracted the last reported value prior to the COVID-19 diagnosis for smoking status and BMI.

| **Table S1C. Comorbidities and Associated ICD-10 Codes.** | |
| --- | --- |
| **Comorbidity** | **ICD-10 Code** |
| Hypertensive diseases | I-10 -I16 |
| Coronary artery disease | I20-I25 |
| Congestive heart failure | I50.* |
| Diabetes | E8-E13 |
| Chronic Respiratory Disease  Asthma  Chronic Obstructive Pulmonary Disease  Emphysema  Obstructive Sleep Apnea | J45.*  J44.*  J43.*  G47.3* |
| Immunosuppression  HIV  History of solid organ transplant | B20.*, Z20.*  Z94.* |
| Kidney Disease  Chronic  End stage | N18.*  N18.6* |
| Chronic Liver Disease  Cirrhosis  Chronic Hep B  Chronic Hep C | K74.6*  B18.1*  B18.2* |
| Cancer | C**.* |
| Polycystic Ovary Disease | E28.2 |

| **Table S1D. Predictors with Associated Clinical Cut Points** | |
| --- | --- |
| **Predictors/Lab values** | **Cut points** |
| A1C | <9 versus ≥9 |
| Blood Glucose | <215 versus ≥215 mg/dL |
| AST | ≤40 versus >40 |
| ALT | ≤40 versus >40 |
| Obesity | 30 > BMI ≥ 30 |

| **Table S1E. Time Between Lab Test and COVID-19 Diagnosis** | | | |
| --- | --- | --- | --- |
| **Predictors/Lab values** | **Median Number of Days** | **Quartile Range** | **Maximum** |
| A1C | 188 | 276 | 692 |
| Blood Glucose | 130 | 266 | 699 |
| AST | 145 | 308 | 699 |
| ALT | 147 | 306 | 704 |

| Table S1F. ALT Component Names and Procedure Codes. | |
| --- | --- |
| Component Name | Procedure Code |
| ALT (SGPT) | 80053.21 |
| ALT (SGPT) | 80053.39 |
| ALT (SGPT) | 80053.44 |
| ALT (SGPT) | 80053.46 |
| ALT (SGPT) | 80053.48 |
| ALT (SGPT) | 80053.55 |
| ALT (SGPT) | 80053.69 |
| ALT (SGPT) | 80076.11 |
| ALT (SGPT) | 80076.2 |
| ALT (SGPT) | 80076.21 |
| ALT (SGPT) | 80076.22 |
| ALT (SGPT) | 80076.25 |
| ALT (SGPT) | 80076.5 |
| ALT (SGPT) | 81599.08 |
| ALT (SGPT) | 82040.84 |
| ALT (SGPT) | 84460.09 |
| ALT (SGPT) | 84460.12 |
| ALT (SGPT) | 84460.13 |
| ALT (SGPT) | 84460.14 |
| ALT (SGPT) | 84460.31 |
| ALT (SGPT) | 86038.53 |

| Table S1F. ALT Component Names and Procedure Codes. | |
| --- | --- |
| Component Name | Procedure Code |
| AST (SGOT) | 80053.21 |
| AST (SGOT) | 80053.39 |
| AST (SGOT) | 80053.44 |
| AST (SGOT) | 80053.46 |
| AST (SGOT) | 80053.48 |
| AST (SGOT) | 80053.55 |
| AST (SGOT) | 80053.69 |
| AST (SGOT) | 80076.11 |
| AST (SGOT) | 80076.2 |
| AST (SGOT) | 80076.21 |
| AST (SGOT) | 80076.22 |
| AST (SGOT) | 80076.25 |
| AST (SGOT) | 80076.5 |
| AST (SGOT) | 82040.84 |
| AST (SGOT) | 84450.08 |
| AST (SGOT) | 84450.12 |
| AST (SGOT) | 84450.13 |
| AST (SGOT) | 84450.14 |
| AST (SGOT) | 84450.3 |
| AST (SGOT) | 86038.53 |

**List of References**

1. Centers for Disease Control and Prevention. *Science Brief: Evidence Used to Update the List of Underlying Medical Conditions Associated with Higher Risk for Severe COVID-19.* 2022 June 15, 2022 [cited 2022 11/11/2022]; Available from: <https://www.cdc.gov/coronavirus/2019-ncov/science/science-briefs/underlying-evidence-table.html>.

2. Joffe, M.M. and P.R. Rosenbaum, *Invited commentary: propensity scores.* Am J Epidemiol, 1999. **150**(4): p. 327-33.

3. Rosenbaum, P.R. and D.B. Rubin, *Constructing a control group using multivariate matched sampling methods that incorporate the propensity score.* Am Stat, 1985. **39**(1): p. 33-38.
