# Supplementary material for: Targeting patients for early COVID-19 therapy; Pre-infection metabolic dysfunction, polycystic ovary syndrome and risk of severe disease in patients under 65: A Massachusetts community-based observational study
Source: PLoS One. 2023 Jun 15;18(6):e0287430. doi: 10.1371/journal.pone.0287430 (PMC10270632; doi:10.1371/journal.pone.0287430)
Supplement: S2 Appendix — (DOCX) [file pone.0287430.s002.docx]

**S2 Appendix: Model Composition and Sensitivity Analyses**

| **Table S2A. Propensity Score Model Composition** | | | |  |  |  |
| --- | --- | --- | --- | --- | --- | --- |
|  | **All** |  |  | **Under 65** |  |  |
| **exposure** | **OR** | **CI** | **removed terms** | **OR** | **CI** | **removed terms** |
| ALT | 1.74 | (1.31, 2.31) | None | 1.62 | (1.09, 2.40) | sex*chf, artery*chf, hyper*chf |
| AST | 1.98 | (1.52, 2.57) | None | 1.94 | (1.18, 3.19) | renal*chf artery*chf hyper*chf not_white*chf chron_resp*chf hisp_unk*chf renal*hyper renal*artery chron_resp*artery diabetes*artery diabetes*renal not_white*renal age*chf bp_systolic*chf |
| glucose | 1.55 | (1.08, 2.23) | diabetes*not_white, diabetes*renal, diabetes*artery diabetes*chf | 2.42 | (1.29, 4.56) | diabetes*not_white, diabetes*renal, diabetes*artery, diabetes*chf, chf*hisp_unk, chf*chron_resp, chf*hyper |
| A1C | 1.34 | (0.82, 2.17) | renal*hyper, diabetes, diabetes*renal evr_smoke*diabetes diabetes*chron_resp diabetes*artery diabetes*chf | 2.31 | (1.14, 4.66) | diabetes, all diabetes interactions, renal*chf, artery*chf, renal*artery, not_white*chf, sex*renal, chron_resp*chf, not_white*artery, hisp_unk*chron_resp, renal*hyper, chron_resp*renal, chron_resp*artery, hisp_unk*chf, hisp_unk*artery, sex*artery, artery*hyper, evr_smoke*chf, hisp_unk*renal, age*chron_resp, sex*evr_smoke, age*evr_smoke |
| Full model includes 12 main effects and all two-way interactions of these main effects: age, diabetes, BMI, sex, ever_smoke, hyper (hypertension), chf (congestive heart failure), artery (arterial disease), renal (chronic renal disease), not_white (race), hisp_unk (ethnicity), and bp_systolic (systolic blood pressure) | | | | | | |

**Sensitivity Analysis**

| **Table S2B. All Ages: Pre-infection Exposure Characteristics and Risk of Severe COVID-19 in PCR Positive Patients Only** | | |
| --- | --- | --- |
| **Exposures*** | **Severe COVID-19**** |  |
|  | **Odds Ratio** | **95% Confidence Interval** |
| Blood Glucose High ( >=215 mg/dL) All ages | OR=1.73 | (1.16, 2.57) |
| ALT > 40 All Ages | OR=1.79 | (1.32, 2.42) |
| AST >40 All Ages | OR=2.09 | (1.57, 2.77) |
| *Model for A1C high (>=9) All ages did not converge. ** Severe COVID-19- defined as COVID-19 positive patients who had a COVID-related hospitalization or death. Each exposure was matched (using Greedy Matching) to up to three unexposed subjects having the closest available propensity scores, without replacement. | | |
